# Effects of 24 Weeks of a Supervised Walk Training on Knee Muscle Strength and Quality of Life in Older Female Total Knee Arthroplasty: A Retrospective Cohort Study

**DOI:** 10.3390/healthcare11030356

**Published:** 2023-01-26

**Authors:** Wei-Hsiu Hsu, Wei-Bin Hsu, Zin-Rong Lin, Shr-Hsin Chang, Chun-Hao Fan, Liang-Tseng Kuo, Wen-Wei Robert Hsu

**Affiliations:** 1 Sports Medicine Center, Chang Gung Memorial Hospital, No. 6, West Section, Chia-Pu Road, Chia-Yi County, Pu-Tz City 61363, Taiwan; 2 Department of Orthopaedic Surgery, Chang Gung Memorial Hospital, No. 6, West Section, Chia-Pu Road, Chia-Yi County, Pu-Tz City 61363, Taiwan; 3 School of Medicine, College of Medicine, Chang Gung University, No. 259, Wenhua 1st Road, Guishan District, Taoyuan City 33302, Taiwan; 4 Department of Athletic Sports, National Chung Cheng University, No. 168, University Road, Minhsiung Township, Chia-Yi County 62102, Taiwan

**Keywords:** total knee arthroplasty, walk training, knee muscle strength, quality of life

## Abstract

Poor supervision, impaired exercise adherence, and low compliance with exercise regimens result in inconsistent effects regarding exercise interventions. A supervised-walk training regimen (9 km/week) may have a positive effect on functional recovery in female total knee arthroplasty (TKA). This study aimed to evaluate the effect of a supervised walking regimen on lower limb muscle strength, functional fitness, and patient-reported outcomes in female TKA. Twenty-eight female TKA were allocated into a control (CON) (*n* = 14) or walk training (WT) (*n* = 14) group. WT on treadmills was initiated 12 weeks after TKA. All patients were examined for lower muscle strength (including extension and flexion of hip and knee), physical function (including a 6-min walk test, 8-foot up-and-go test, and 30-s chair stand test), and Knee Injury and Osteoarthritis Outcome Score (KOOS) questionnaire. Knee flexor (WT: CON; 64.4 ± 4.1 nm/kg: 43.7±3.3 nm/kg; *p* = 0.001; effect size: 5.62) and extensor strengths (WT: CON; 73.1 ± 7.5 nm/kg: 48.2 ± 2.4 nm/kg; *p* = 0.001; effect size: 4.47) statistically increased in the WT group compared to the CON group. The 6-min walk test (from 341.3 ± 20.5 m to 405.5 ± 30.7 m; *p* = 0.001; effect size: 2.46) and 8-foot up-and-go test (from 9.5 ± 0.7 s to 8.3 ± 0.7 s; *p* = 0.002; effect size: 1.71) tests also showed significant improvements in the WT group in the follow-up compared to the baseline. An increase in quality of life score according to the KOOS questionnaire (WT: CON; 91.0 ± 2.8: 68.1 ± 5.8; *p* = 0.001; effect size: 5.02) was noted in the WT group compared to the CON group in the follow-up. WT facilitated improvements in knee muscle strength and functional outcomes in TKA patients.

## 1. Introduction

Total knee arthroplasty (TKA) is a well-established knee reconstruction procedure for treating advanced osteoarthritis (OA) and a reliable surgical procedure for alleviating pain, accelerating functional recovery, and improving quality of life (QoL) with respect to patient-reported outcomes [1,2]. The number of patients undergoing TKA has steadily increased every year, and primary TKA patients aged <65 years are estimated to comprise more than half of all TKA patients in the United States [3]. Postoperative rehabilitation following TKA is an important issue. A decrease in the strength of both the quadriceps and hamstring occurs following TKA. This decline in the muscle strength of the lower limbs is associated with a decline in functional outcomes [4,5]. Postoperative rehabilitation aims to increase the range of motion of the knee, muscle strength, and proprioceptive functions after TKA. Various exercise interventions have been associated with different levels of positive effects in patients after TKA [6,7,8,9]; however, these effects are inconsistent because of poor supervision, impaired exercise adherence, and low compliance with exercise regimens [10,11,12].

Walking is the most common and essential physical activity of daily living (ADL). It is also a complex activity involving all levels of the neuromuscular and cardiorespiratory systems and has numerous health benefits [13]. Meanwhile, walking training has a positive effect on the health-related and perceived quality of Life in older persons [14,15]. Thus, we performed an easy training regimen, walk training (WT), on patients in TKA recovery for the purpose of reducing the bias of poor supervision. Because the number of female patients who had undergone TKA was approximately twice that of men between 2001 and 2010 [16], this study focused only on female patients who had undergone TKA. Additionally, patient-reported outcome measurements are routinely used in clinical research for assessing patients’ health statuses [17]. The Knee Injury and Osteoarthritis Outcome Score (KOOS) questionnaire is the most reliable and relevant patient-reported outcome measurement to assess knee-specific and related problems, including patients’ quality of life [18]. Therefore, we used the KOOS questionnaire to evaluate the health status of TKA. This study aimed to evaluate the effect of a supervised walking regimen on lower limb muscle strength, functional fitness, and patient-reported outcomes in female patients with TKA. We hypothesized that the WT would have positive effects on low limb muscle strength, performance on the functional fitness test, and KOOS in female TKA.

## 2. Materials and Methods

### 2.1. Participants

The retrospective cohort study was approved by the Ethics Committee and Institutional Review Board (IRB: 102-0979B, Approval Date: 26 April 2013). The participants were recruited in the orthopedic clinic of the hospital, and the orthopedic physician explained the protocol to the participants. All patients provided informed consent. The eligibility criteria were female patients aged 60~80 who had been diagnosed with Ahlbäck stage III-IV knee OA [19] without infectious joint disease or rheumatic disease, and who had undergone TKA without additional surgeries such as ligament reconstruction or corrective osteotomy. Patients with diabetes, a neuromusculoskeletal disorder, history of fracture of the lower limb, or the presence of an artificial limb, as well as those who were unsuitable for exercise training, were excluded.

The participants were allocated into two groups by their willingness. Patients in the control (CON) and WT groups followed the routine postoperative rehabilitation protocol, including a self-administered straight leg raise (in a sitting position, static stretching exercises for hamstrings and gastrosoleus muscles), and a range of motion exercise consisting of terminal knee extension with weight; straight leg raises with weight in the supine and side-lying positions; and prone, hip, and knee flexion–extension with weight in supine as well as knee flexion–extension with weight in prone. Moreover, participants in the WT group practiced an extra walking intervention for 24 weeks.

WT was started 3 months after TKA, because patients could tolerate exercise training as the pain had notably decreased at this time [8]. All assessments in both the CON and WT groups were performed at the following time points (Supplementary Figure S1): preoperatively, 12 weeks postoperatively (baseline), 12 weeks after the start of training (mid-exercise), after training (post-exercise), and 12 weeks after completing the training (follow-up). All assessments in both groups were conducted on the same day by the same experienced investigator, who was blinded to the participant allocation, at the Sports Medicine Center.

**Figure 1:**
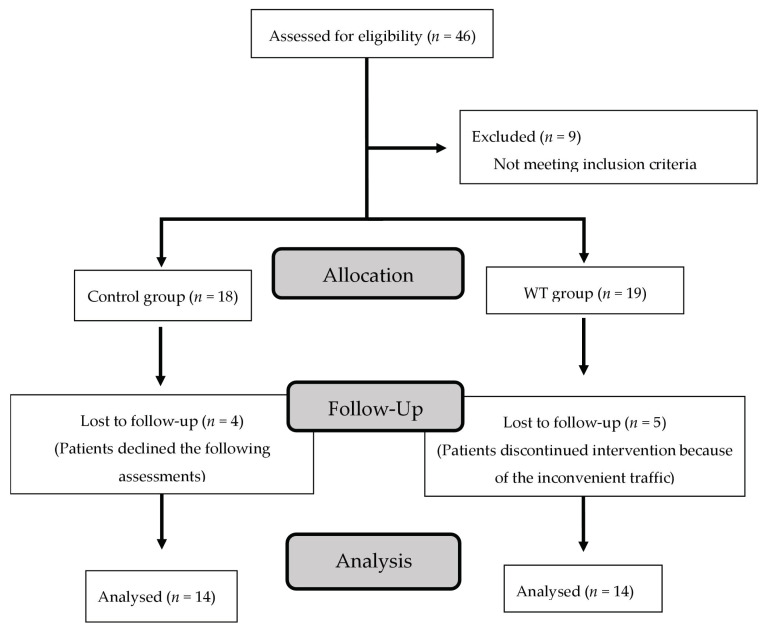
Flowchart of patient enrollment.

From August 2015 to August 2017, 46 female patients with end-stage OA were identified from the author’s clinics. Among these, nine patients were excluded because they did not meet the inclusion criteria. In total, 37 female participants were enrolled. After the initial allocation, there were 18 patients in the CON group and 19 patients in the WT group. Because four patients in the CON group declined the following assessments and five patients in the WT group discontinued the intervention, nine patients were excluded from the final analysis. Thus, 28 patients (14 each in the CON and WT groups) completed the treatment regimen and were included in the final analysis (Figure 1; the template of the flow chart was adopted from the reference [20]). 

### 2.2. Sample Size

The sample size was determined using G*power software version 3.1.9.7. (Heinrich Heine University, Dusseldorf, Germany). The input parameters used for the t test were alpha = 0.05, effect size d = 0.8, and the power was calculated as 0.8. The total sample size was calculated to be 30 participants, with fifteen participants in each group. In previous patients’ surveys, most patients were divided into different groups, with the number of people in each group ranging from 14 to 20 for statistical analysis [7,8,21]. The intraclass correlation coefficient (ICC), typical error (TE), and percentual coefficient of variation (CV) were calculated to access intertest correlation (Supplementary Table S1).

### 2.3. WT Course

Schimpl and his colleagues reported that the average walking speed of elders aged over 60 years is about 1.2 m/s (~4.32 km/h) [22]. Additionally, Serwe and his colleagues reported that the effective duration (30 minutes/day × 5 days) of walk training for increasing physical activity in women is a total of 2.5 hours a week [23]. Thus, a total distance of 10.8 km (4.32 km/h × 2.5 h = 10.8 km) per week might be an effective prescript of walk training for healthy women. Considering the patients’ baseline 6-minute walk testing results (average walking speed about 3.4 km/h in the two groups), a total distance of 8.5 km (3.4 km/h × 2.5 h = 8.5 km) a week was determined to be a more effective prescript of walk training for female TKA patients. Therefore, we determined that the training target was to finish a total of 9 km a week. The participants in the WT group were asked to complete a total distance of 9 km a week on the treadmill with unlimited walking speed, at an intensity of 50–70% of the target heart rate, monitored by a POLAR FT40 monitor (Polar Electro Oy, Kempele, Finland). The walking speed was dependent on the individual, and the entire training process was recorded and supervised by a trained physical therapist at the Sports Medicine Center. Maximum heart rate (100% target heart rate) was calculated using the 220-age formula [24]. During the training period, the patients’ motion and physical conditions were supervised by at least one trained physical therapist for the WT group. To determine the distance and reduce injury risk from the environment, such as uneven or sloping ground, WT was performed using the JOHNSON Matrix T50X treadmill (Johnson Health Tech, Taichung, Taiwan).

### 2.4. Muscle strength

The isokinetic dynamometer HUMAC NORM system (CSMi^®^, Stoughton, MA, USA) was used to assess the lower extremity muscle strength of the affected leg, including the extension and flexion of the hip and knee [7]. The muscle strength of the hip was evaluated when participants were in the supine position, and that of the knee was tested in the seated position. Angular velocity of 60° per second was set in the concentric contraction model. Isokinetic tests were performed five times for each examination, and the maximum force was recorded. There was a 3-min rest period between hip and knee trials. Body composition is associated with muscle strength, especially fat-free mass. To avoid the influence of body composition, the value of muscle strength was normalized by body weight. Normalized force is an effective and useful method to compare persons of different individual sizes [25].

### 2.5. Functional Fitness Test

The 6-minute walk test (6MWT), the 8-foot up-and-go test (8UG), and the 30-second chair stand test (30CST) were used to assess exercise capacity, motor agility, and lower-body muscle strength, respectively. The details of these tests, such as criterion-referenced standard, validity, and reliability, have been described in a previous study [26]. The procedure of 6MWT is to measure the distance that the subject can walk over a total of six minutes on a hard and flat surface. The protocol of the 8UG is to record the time that the subjects are asked to rise from a chair placed next to a wall, walk to a traffic cone at 8 feet in front of the chair, turn, walk back, and sit down. The 30CST is intended to record the number of times the subjects stand up with a straight back and hands on the opposite shoulder crossed at the wrists from a chair without armrests in 30 seconds.

### 2.6. Knee Injury and Osteoarthritis Outcome Score (KOOS) Questionnaire

Clinical knee scoring was used to assess outpatients’ self-reported outcomes by using the Chinese version of the KOOS questionnaire [27]. KOOS contains forty-two items in the five subscales, named Symptoms (seven items), Pain (nine items), Activity of daily livings (ADL; seventeen items), Sport (five items), and knee-related quality of life (QoL; four items). All items have five possible answer options presented with a Likert scale. The scores range from 0 (Good) to 4 (Worse). Each subscale score is calculated using $100-\frac{Mean\;score\;\left(All\;items\;in\;each\;subscale\right)\;\times \;100}{4}$ formulas, and scores are transformed to a 0–100 scale. A zero score indicates an extreme problem. 

### 2.7. Statistical Analysis

All data were analyzed using the Statistical Package for the Social Sciences, Windows version 17.0 (SPSS^®^, Chicago, IL, USA). The difference in demographic data between the two groups was tested by the unpaired Student’s *t*-test. Generalized estimating equations (GEEs) [28] were used to resolve the differences of each assessment outcome between the WT and CON groups, as well as within the groups. The Shapiro–Wilk results showed no abnormal distributions in either group in pre-operation (Supplementary Table S2). The effect size (ES) was used to measure the difference between group means. The relative ES was calculated using Cohen’s d, which is defined as the difference between two means divided by a standard deviation for the data. The primary outcome of the study was the QoL subscale of KOOS. The effect size between the CON and WT groups at follow-up was 5.02, which indicates a huge effect size [29]. The ideal power was considered to be 80%, with a 0.05 type I error. All continuous data are presented as the mean ± standard deviation, and *p*-values of <0.05 were considered statistically significant. To reduce bias, we attempted to identify controls with matching ages and body mass indices.

## 3. Results

The patients’ demographic data are shown in Table 1. The mean age was 68.5 ± 6.6 and 69.7 ± 2.8 years in the CON and WT groups, respectively. Height and weight were similar between the CON and WT groups. Lower extremity muscle strength, functional test results, and KOOS scores were similar between the CON and WT groups (*p* > 0.05).

In the lower extremity muscle strength assessment, the WT group exhibited a significant increase in hip extensor, knee extensor, and knee flexor muscle strengths compared to the CON group at the post-exercise assessment, and the differences in knee extensor and flexor muscle strengths lasted until the follow-up (Table 2). When participants were compared within each group in a temporal manner (Table 3), the CON group exhibited a significant increase only in knee extensor muscle strength at the final follow-up compared with the baseline measurement. However, the WT group exhibited a significant increase in hip extensor, knee extensor, and knee flexor measurements at the post-exercise assessment compared with the baseline. The knee extensors even exhibited a significant increase in muscle strength during the mid-exercise assessment.

The functional outcomes of the functional fitness tests (Table 4) revealed that the results of the 6MWT, 8UG, and 30CST did not differ significantly between the CON and WT groups at each assessment time point. However, in the temporal comparison (Table 5), the results of the 6MWT and 8UG showed significant improvements at the mid-exercise, post-exercise, and follow-up measurements compared with baseline measurements in the WT group, but not in the CON group. 

The self-explanatory KOOS questionnaire revealed (Table 6) that the QoL subscale was significantly increased in the WT group compared to the CON group at follow-up. When a temporal comparison was performed within each group (Table 7), scores for the subscales of symptoms, pain, and ADL exhibited significant improvements in both the CON and WT groups at the follow-up compared with the baseline. The improvement in scores in the pain and ADL subscales in the WT group occurred at an earlier time point (pain scores decreased at the post-exercise evaluation; ADL scores increased at the mid-and post-exercise evaluations). Among these subcategories, scores for the QoL subscale in the WT group exhibited a significant increase in the post-exercise and follow-up assessments compared with the baseline; however, these improvements were not observed in the CON group.

## 4. Discussion

The most important findings of the present study were the statistical increase in the QoL subcategory of KOOS and the knee muscle strength (extensors and flexors) in the WT group compared to the CON group at follow-up (Table 2 and Table 6). While the comparison was performed within the individual group in a temporal manner (Table 3 and Table 5), the knee extensors and functional fitness test (6 MWT: ambulatory capacity, 8UG: motor agility) in the WT group were improved at the mid-exercise, post-exercise, and follow-up measurements compared with the baseline, suggesting that WT enhanced the recovery of functional mobility after TKA [30,31,32,33]. These increases were based on the concomitant improvements in the pain, symptoms, and ADL subcategories of KOOS after TKA, quadriceps training, and range of motion exercises [10,34,35]. In addition to the range of motion of knee and muscle strength on which the TKA standard exercises focus, WT may provide extra endurance and proprioception.

Many studies have shown that an increase in the quadriceps strength of TKA patients is associated with superior outcomes and performance [36,37,38,39,40]. Previous studies have also revealed that muscle strengthening improves quadriceps strength and functional fitness test scores (timed up-and-go, stair-climbing test, and 6MWT) [41,42]. Our results were similar to other findings in the literature, and showed that the knee extensor and flexor strength in the WT group significantly improved compared with the CON group (Table 2). In the temporal comparison within individual groups, the knee extensor improvement in the WT group occurred simultaneously with the improvements in the 6MWT and 8UG functional tests (Table 4 and Table 5), as well as in the ADL domains of KOOS (Table 6 and Table 7). These results imply that lower leg muscle strength is a determinant physical factor and plays a pivotal role in functional outcomes in TKA patients [43,44]. However, no significant difference in the 6MWT, 8UG, or the ADL subscales of KOOS was observed between the two groups. The lack of a significant difference might have resulted from the small sample size.

All patients in the WT group were supervised by a trained physical therapist during training, and no patient in the WT group reported discomfort or needed further medical management. Our results suggest that WT is beneficial for patients who have undergone TKA. Considering the ease of standardization and performance of WT, it can be effectively applied during rehabilitation following TKA in community-dwelling patients. However, a trained physical therapist also plays an important role in the intervention. In this study, a therapist monitored the status of the participants and gave real-time assistance when the participants had an issue during the exercise. Therefore, the deployment of a trained physical therapist would be recommended.

Several limitations of this study should be acknowledged. First, evidence regarding the entire effect of WT in TKA was restricted because of the small sample size in this retrospective study. Nevertheless, improvements in muscle strength and function over time indicated the benefits of WT in TKA. Second, our results (12 months after surgery) did not fully demonstrate the long-term effects of WT. The results showed that multiple metrics gradually improved in the CON group. Third, there was no random allocation, and certain participants could be more motivated, which could influence the results. Thus, a larger randomized controlled trial is needed to verify the findings of this study, as it was limited to only 28 who completed the study out of 46 total patients. Fourth, neuromuscular activation also plays a critical role in muscle function. Electromyography of the lower limbs might be included for greater clarity regarding the effect of WT on the neuromuscular system in further study. 

## 5. Conclusions

During WT, no patient in the WT exercise group reported discomfort or injury or required further medical management. The 9 km/week WT protocol is feasible for TKA patients. The WT could significantly improve muscle strength (knee flexion and extension), functional outcome (6WT and 8UG), and QoL of patients. The simplicity of WT could reduce the limitations of poor supervision, impaired exercise adherence, and low compliance with exercise regimens, which cause poor rehabilitation outcomes. The authors believe that WT should be broadly applied in the rehabilitation of patients who have undergone TKA.

## Figures and Tables

**Table 1:** The demographic characters of patients at the pre-operative assessment.

Characters	CON (*N* = 14)	WT (*N* = 14)	*p*	ES
Age (years)	68.5 ± 6.6	69.7 ± 2.8	0.372	0.23
Height (cm)	152.0 ± 5.8	152.8 ± 4.9	0.877	0.14
Weight (kg)	65.9 ± 11.5	66.4 ± 9.8	0.682	0.04
BMI (kg/m^2^)	28.4 ± 4.0	28.4 ± 3.4	0.567	0.01
	Pre-operation			
Muscle strength				
HE (Nm/kg)	72.0 ± 8.2	79.0 ± 9.2	0.571	0.80
HF (Nm/kg)	32.4 ± 3.5	31.3 ± 4.1	0.844	0.28
KE (Nm/kg)	42.1 ± 2.9	41.5 ± 5.3	0.941	0.14
KF (Nm/kg)	41.8 ± 3.0	45.2 ± 2.4	0.376	1.25
Functional test				
6MWT (m)	309.1 ± 31.4	326.1 ± 29.7	0.693	0.55
8UG (sec)	9.5 ± 1.6	10.7 ± 1.3	0.541	0.82
30s-CST (Times)	10.6 ± 1.1	10.4 ± 0.9	0.836	0.19
KOOS				
Sym	63.9 ± 5.1	63.7 ± 6.3	0.983	0.03
Pain	65.6 ± 4.8	63.8 ± 5.6	0.817	0.31
ADL	64.5 ± 4.9	68.4 ± 6.2	0.611	0.69
Sports	40.6 ± 7.1	35.5 ± 4.4	0.550	0.86
QoL	61.1 ± 5.4	51.5 ± 5.8	0.218	1.71

Data are presented as mean ± SD. Abbreviations: CON: control; WT: walk training; HE: hip extension; HF: hip flexion; KE: knee extension; KF: knee flexion; 6MWT: 6-min walk test; 8UG: 8-foot up-and-go; 30 s-CST: 30-s chair stand test; Sym: symptom; ADL: activity of daily living; QoL: quality of life; ES: effect size. There was no difference between the two groups.

**Table 2:** Comparison of muscle strength between two groups of TKA patients.

(Nm/kg)	Baseline				Mid-Exercise				Post-Exercise				Follow-Up			
	CON	WT	ES	*p*	CON	WT	ES	*p*	CON	WT	ES	*p*	CON	WT	ES	*p*
HE	71.5 ± 5.4	85.9 ± 9.9	1.58	0.199	74.6 ± 9.6	102.6 ± 12.5	2.51	0.074	77.5 ± 8.0	116.9 ± 11.3 §	4.02	0.004 *	87.6 ± 10.6	106.8 ± 9.4 §	1.91	0.177
HF	27. 6 ± 2.8	33.4 ± 3.6	1.79	0.190	26.4 ± 4.3	34.5 ± 4.2	1.90	0.172	27.4 ± 3.6	37.1 ± 4.7	2.32	0.094	29.6 ± 3.1	33.9 ± 5.4	0.97	0.452
KE	40.3 ± 5.1	45.2 ± 4.9	1.17	0.476	45.9 ± 4.1	57.6 ± 5.7 §	2.36	0.091	48.2 ± 2.4	73.1 ± 7.5 §	4.47	0.001 *	54.2 ± 3.2 §	71.4 ± 6.6 §	3.31	0.019 *
KF	43.6 ± 4.0	46.1 ± 3.0	0.70	0.615	49.8 ± 3.6	54.1 ± 4.0	1.13	0.406	43.7 ± 3.3	64.4 ± 4.1 §	5.62	0.001 *	43.1 ± 3.0	65.0 ± 2.6 §	1.27	0.001 *

Data are presented as mean ± SD. *: A significant difference between two groups. §: Significant difference compared to baseline within the individual group (*p* < 0.05). Abbreviations: CON: control; WT: walk training; HE: hip extension; HF: hip flexion; KE: knee extension; KF: knee flexion; ES: effect size. There were significant differences in knee extension and flexion between the two groups.

**Table 3:** The effect sizes and *p*-values of comparison of muscle strength between different time points and baselines within the WT and CON groups.

		ES			*p*		
Variable		Mid-Exercise	Post-Exercise	Follow-Up	Mid-Exercise	Post-Exercise	Follow-Up
WT	HE	1.48	2.92	2.17	0.211	0.002	0.024
	HF	0.28	0.88	0.11	0.751	0.514	0.154
	KE	2.33	4.40	4.51	0.032	0.001	0.012
	KF	2.26	5.09	6.73	0.301	0.002	0.001
CON	HE	0.40	0.88	1.91	0.468	0.216	0.165
	HF	0.33	0.06	0.68	0.532	0.151	0.205
	KE	1.21	1.98	3.26	0.541	0.304	0.023
	KF	1.63	0.03	0.14	0.665	0.501	0.487

Abbreviations: CON: control; WT: walk training; HE: hip extension; HF: hip flexion; KE: knee extension; KF: knee flexion; ES: effect size.

**Table 4:** Comparison of physical function fitness between the two groups of TKA patients.

	Baseline				Mid-Exercise				Post-Exercise				Follow-Up			
	CON	WT	ES	*p*	CON	WT	ES	*p*	CON	WT	ES	*p*	CON	WT	ES	*p*
6MWT (m)	331.0 ± 16.5	341.3 ± 20.5	0.55	0.696	346.6 ± 14.8	375.2 ± 21.4 §	1.55	0.271	346.1 ± 16.8	400.1 ± 23.8 §	2.62	0.40	345.8 ± 16.5	405.5 ± 30.7 §	2.42	0.093
8UG (sec)	10.4 ± 0.7	9.5 ± 0.7	1.28	0.386	9.1 ± 0.5	8.5 ± 0.5 §	1.20	0.427	9.1 ± 0.8	8.6 ± 0.8 §	0.62	0.692	9.2 ± 0.6	8.3 ± 0.7 §	1.38	0.302
30s-CST (Times)	11.9 ± 0.9	12.2 ± 0.7	0.37	0.764	12.6 ± 0.6	14.0 ± 1.0	1.69	0.238	12.6 ± 0.8	14.21 ± 1.0	1.76	0.211	12.8 ± 0.7	14.0 ± 1.2	1.22	0.370

Data are presented as mean ± SD. §: Significant difference compared to baseline within the individual group (*p* < 0.05). Abbreviations: CON: control; WT: walk training; 6MWT: 6-minute walk test; 8UG: 8 feet up-and-go; 30s-CST: 30-second chair stand test; ES: effect size. 6MWT and 8UG tests were statistically improved after WT in female TKA patients at the post-exercise and follow-up evaluations.

**Table 5:** The effect sizes and *p*-values of comparison of physical function fitness between different time points and baselines within the WT and CON groups.

		ES			*p*		
Variable		Mid-Exercise	Post-Exercise	Follow-Up	Mid-Exercise	Post-Exercise	Follow-Up
WT	6MWT	1.62	2.65	2.46	0.031	0.012	0.001
	8UG	1.64	1.20	1.71	0.029	0.031	0.002
	30s-CST	2.09	2.33	1.83	0.376	0.471	0.389
CON	6MWT	0.96	0.90	0.85	0.588	0.546	0.437
	8UG	2.14	1.73	1.84	0.632	0.652	0.594
	30s-CST	0.92	0.82	1.12	0.417	0.374	0.360

Abbreviations: CON: control; WT: walk training; 6MWT: 6-minute walk test; 8UG: 8 feet up-and-go; 30s-CST: 30-second chair stand test; ES: effect size.

**Table 6:** Comparison of Knee Injury and Osteoarthritis Outcome Score (KOOS) subscales between two groups of TKA patients.

		Baseline				Mid-Exercise			Post-Exercise				Follow-Up			
	CON	WT	ES	*p*	CON	WT	ES	*p*	CON	WT	ES	*p*	CON	WT	ES	*p*
Sym	62.1 ± 4.9	71.1 ± 5.4	1.74	0.200	71.2 ± 5.7 §	76.0 ± 4.7	0.91	0.487	70.9 ± 4.9	78.2 ± 3.9	1.64	0.253	83.0 ± 4.1 §	86.7 ± 3.8 §	0.93	0.514
Pain	78.2 ± 3.4	79.5 ± 4.4	0.33	0.794	84.8 ± 3.5	87.6 ± 4.0	0.74	0.584	84.3 ± 4.3	90.5 ± 2.0 §	1.84	0.185	91.1 ± 3.1 §	97.3 ± 1.5 §	2.54	0.072
ADL	76.3 ± 3.2	76.6 ± 4.7	0.07	0.923	80.2 ± 5.1	89.8 ± 2.4§	2.40	0.083	78.7 ± 5.1	87.6 ± 2.6 §	2.19	0.118	92.0 ± 2.5 §	94.6 ± 1.3 §	1.30	0.322
Sports	47.0 ± 5.2	42.3 ± 2.8	1.12	0.439	43.6 ± 3.8	50.5 ± 4.5	1.65	0.229	45.1 ± 4.7	44.0 ± 2.8	0.28	0.835	45.2 ± 3.8	45.6 ± 4.9	0.09	0.936
QoL	56.6 ± 4.7	60.0 ± 6.3	0.61	0.696	64.6 ± 7.3	71.4 ± 6.0	1.01	0.474	63.4 ± 5.8	72.8 ± 4.3 §	1.84	0.193	68.1 ± 5.8	91.0 ± 2.8 §	5.02	0.001 *

Data are presented as mean ± SD. *: A significant difference between two groups. §: Significant difference compared to baseline within the individual group (*p* < 0.05). Abbreviations: CON: control; WT: walk training; Sym: symptom; ADL: activity of daily livings; QoL: quality of life; ES: effect size. The scores of the QoL subscale in the WT group exhibited a significant increase in the post-exercise and follow-up assessments compared with the baseline.

**Table 7:** The effect sizes and *p*-values of comparison of KOOS subscales between different time points and baselines within the WT and CON groups.

		ES			*p*		
Variable		Mid-Exercise	Post-Exercise	Follow-Up	Mid-Exercise	Post-Exercise	Follow-Up
WT	Sym	0.97	1.51	3.34	0.521	0.321	0.002
	Pain	1.93	3.22	5.42	0.074	0.024	0.002
	ADL	3.54	2.90	5.22	0.032	0.008	0.002
	Sports	2.19	0.61	0.83	0.658	0.713	0.072
	QoL	1.85	2.37	6.36	0.067	0.012	0.001
CON	Sym	1.71	1.80	4.63	0.047	0.051	0.035
	Pain	1.91	1.57	3.97	0.514	0.624	0.041
	ADL	0.92	0.56	5.47	0.727	0.694	0.037
	Sports	0.75	0.38	0.40	0.865	0.792	0.698
	QoL	1.30	1.29	2.18	0.476	0.523	0.331

Abbreviation: CON: control; WT: walk training; Sym: symptom; ADL: activity of daily livings; QoL: quality of life; ES: effect size.

## Data Availability

Remaining data are available from the corresponding author upon reasonable request.

## Supplementary Materials

The following supporting information can be downloaded at: https://www.mdpi.com/article/10.3390/healthcare11030356/s1, Figure S1: Schematic diagram of the experimental time course; Table S1: The coefficient of validity and reliability in the WT and CON groups; Table S2: Shapiro–Wilk test of normality.
