# Supplementary material for: Effects of 24 Weeks of a Supervised Walk Training on Knee Muscle Strength and Quality of Life in Older Female Total Knee Arthroplasty: A Retrospective Cohort Study
Source: Healthcare (Basel). 2023 Jan 26;11(3):356. doi: 10.3390/healthcare11030356 (PMC9914605; doi:10.3390/healthcare11030356)
Supplement: Supplementary file 1 [file healthcare-11-00356-s001.zip › healthcare-2100948-supplementary.pdf]

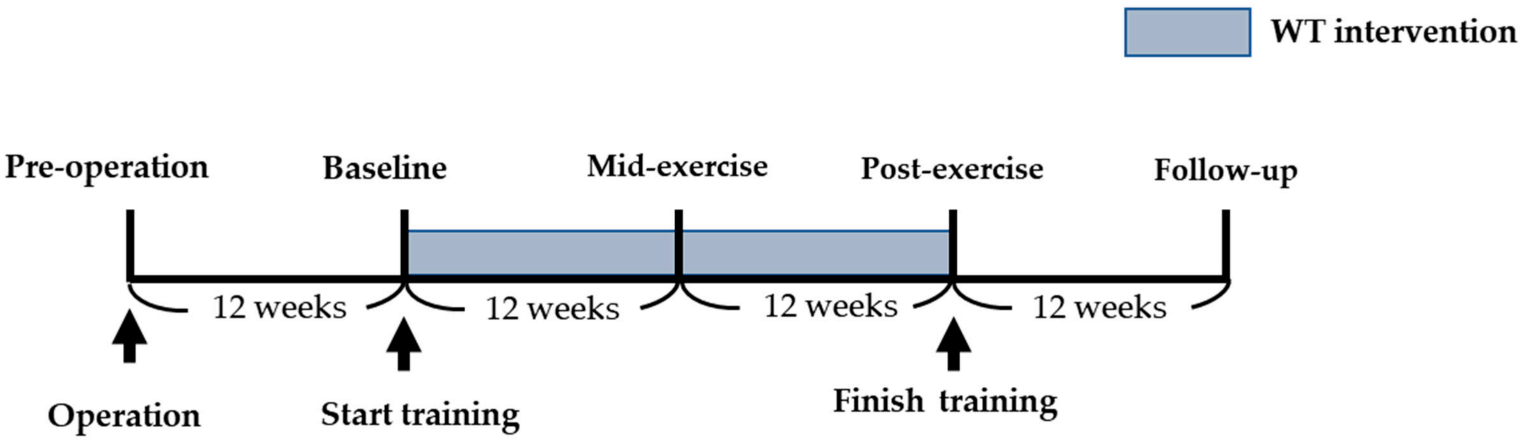

Supplementary Figure S1. Schematic diagram of the experimental time course.

Supplementary Table S1. The coefficient of validity and reliability in the WT and CON groups.

	Variable	Coefficient of variation (%)				Typical error			Intraclass correlation coefficient
		Baseline	Mid-exercise	Post-exercise	Follow-up	Between mid-exercise and baseline	Between post- and mid-exercise	Between follow-up and post-exercise	
WT	HE	45	47	37	33	5	5	14	0.573
	HF	41	48	49	63	3	3	9	0.724
	KE	42	39	40	38	4	4	9	0.873
	KF	25	29	25	16	4	3	10	0.525
	6MWT	24	23	24	30	22	5	18	0.897
	8UG	28	21	36	34	5	1	3	0.774
	30s-CST	23	28	27	35	5	1	4	0.837
	Sym	29	25	16	16	13	3	11	0.402
	Pain	20	24	16	13	13	3	9	0.544
	ADL	21	22	8	12	14	4	9	0.448
	Sports	31	35	32	37	15	4	13	0.177
	QoL	38	32	24	20	14	4	14	0.521
CON	HE	30	47	36	37	4	4	12	0.608
	HF	39	62	50	35	3	3	6	0.550
	KE	43	29	19	22	3	3	9	0.474
	KF	35	27	28	22	3	3	7	0.586
	6MWT	19	17	19	18	5	4	14	0.829
	8UG	26	23	35	25	5	1	3	0.613
	30s-CST	31	20	24	21	5	1	2	0.706
	Sym	25	21	25	15	13	4	10	0.385
	Pain	16	15	20	9	11	4	11	0.074
	ADL	15	24	25	7	13	4	11	0.147
	Sports	41	29	39	26	12	3	8	0.472
	QoL	30	40	36	24	17	5	14	0.201

Abbreviation: CON: Control; WT: Walk Training; HE: Hip Extension; HF: Hip Flexion; KE: Knee Extension; KF: Knee Flexion; 6MWT: 6-minutes' walk test; 8UG: 8 feet up and go; 30s-CST: 30-second chair stand test; Sym: Symptom; ADL: Activity of daily livings; QoL: Quality of life.

Supplementary Table S2. Shapiro-Wilk test of normality.

Shapiro-Wilk test		
	Shapiro-Wilk	*p*
Hip Extension	.953	.229
Hip Flexion	.967	.507
Knee Extension	.974	.690
Knee Flexion	.953	.239
6MWT	.952	.345
8-Ft Up-and-Go	.861	.052
30s-CST	.948	.290
Symptom	.909	.094
Pain	.886	.061
Activity of daily livings	.908	.091
Sports	.854	.051
Quality of life	.898	.063
